# Geographical, sex, and socioeconomic differences in non-communicable disease indicators: A cross-sectional survey in Eastern Uganda

**DOI:** 10.1371/journal.pgph.0003308

**Published:** 2024-06-12

**Authors:** Gulam Muhammed Al Kibria, Ankita Meghani, Charles Ssemagabo, Adaeze Wosu, Tryphena Nareeba, Collins Gyezaho, Edward Galiwango, Judith Kaija Nanyonga, George W. Pariyo, Dan Kajungu, Elizeus Rutebemberwa, Dustin G. Gibson

**Affiliations:** 1 Department of International Health, Johns Hopkins University Bloomberg School of Public Health, Baltimore, Maryland, United States of America; 2 Department of Disease Control and Environmental Health, Makerere University College of Health Sciences, Mulago Hill, Kampala, Uganda; Tribhuvan University Institute of Medicine, NEPAL

## Abstract

The prevalence of non-communicable diseases (NCDs) is increasing in many low- and middle-income countries (LMICs). This study examined differences in the burden of NCDs and their risk factors according to geographic, sex, and sociodemographic characteristics in a rural and peri-urban community in Eastern Uganda. We compared the prevalence by sex, location, wealth, and education. Unadjusted and adjusted prevalence ratios (PR) were reported. Indicators related to tobacco use, alcohol use, salt consumption, fruit/vegetable consumption, physical activity, body weight, and blood pressure were assessed. Among 3220 people (53.3% males, mean age: 35.3 years), the prevalence of NCD burden differed by sex. Men had significantly higher tobacco (e.g., current smoking: 7.6% vs. 0.7%, adjusted PR (APR): 12.8, 95% CI: 7.4–22.3), alcohol use (e.g., current drinker: 11.1% vs. 4.6%, APR: 13.4, 95% CI: 7.9–22.7), and eat processed food high in salt (13.4% vs. 7.1, APR: 1.8, 95% CI: 1.8, 95% CI: 1.4–2.4) than women; however, the prevalence of overweight (23.1% vs 30.7%, APR: 0.7, 95% CI: 0.6–0.9) and obesity (4.1% vs 14.7%, APR: 0.3, 95% CI: 0.2–0.3) was lower among men than women. Comparing locations, peri-urban residents had a higher prevalence of current alcohol drinking, heavy episodic drinking, always/often adding salt while cooking, always eating processed foods high in salt, poor physical activity, obesity, prehypertension, and hypertension than rural residents (p<0.5). When comparing respondents by wealth and education, we found people who have higher wealth or education had a higher prevalence of always/often adding salt while cooking, poor physical activity, and obesity. Although the findings were inconsistent, we observed significant sociodemographic and socioeconomic differences in the burden of many NCDs, including differences in the distributions of behavioral risk factors. Considering the high burden of many risk factors, we recommend appropriate prevention programs and policies to reduce these risk factors’ burden and future negative consequences.

## Introduction

Globally, more than 40 million deaths were attributed to non-communicable diseases (NCDs) in 2019 [1]. The proportion of deaths attributed to these conditions (e.g., hypertension, diabetes, obesity, stroke, kidney disease, and other cardiovascular diseases) has increased significantly during the past several decades, especially in low- and middle-income countries (LMICs) [1, 2]. Four modifiable risk factors are primary contributors to this high burden of NCDs—physical inactivity, excessive alcohol use, unhealthy diets, and tobacco consumption [2–5]. Uganda, an LMIC within sub-Saharan Africa, is undergoing epidemiologic and demographic transitions [6–8] and faces a double disease burden, with the prevalence of NCDs increasing alongside an already high burden of communicable diseases [9]. It also has a double nutrition burden with the simultaneous presence of both underweight and overweight/obesity [10, 11]. These epidemiologic and demographic transitions across LMICs like Uganda result from increased urbanization, sedentary lifestyles, and aging populations [7, 12, 13].

Considering the growing burden of NCDs in Uganda and other similar LMICs from sub-Saharan Africa, data from these countries are limited compared to high-income countries (HICs). In sub-Saharan Africa, deaths resulting from infectious diseases have gotten lower while the proportion of deaths attributable to NCDs has increased. When we look into the data from Uganda, the STEPwise Approach to NCD Risk Factor Surveillance (STEPs) 2014 was the first nationwide survey to estimate the prevalence of NCD risk factors in Uganda [14, 15]. That survey indicated that about one-third of adults (i.e., at least 18-year-olds) in Uganda may have more than three NCD risk factors (i.e., physical inactivity, poor quality diet, overweight/obesity, or high blood pressure (BP)). This survey also showed that there are urban-rural disparities in the burden of many NCDs, including higher prevalence of hypertension, diabetes, hypercholesteremia, overweight, and obesity among urban residents. Rural people had a higher burden of several behavioral risk factors like alcohol consumption and tobacco smoking. This was the last available STEPs survey in Uganda; therefore, more recent data are required to estimate the burden of NCDs [16]. Previous studies from Uganda have also documented the determinants of NCDs [8, 11, 17–22]. For instance, Wandera and colleagues reported a higher burden of NCDs (e.g., hypertension, diabetes, and heart diseases) among people with higher family wealth [20]. Kavishe et al. found an association between hypertension and higher education [21]. Despite these, few recent studies have investigated the sociodemographic differences associated with NCD or its behavioral risk factors by comparing people according to major sociodemographic or socioeconomic determinants, especially in rural areas where most people live.

In a peri-urban, resource-limited context like Uganda and other sub-Saharan African countries, where only a tiny proportion of the annual budget is spent on healthcare, differences in health outcomes related to NCDs are particularly pronounced [23, 24]. Factors such as limited access to healthcare services, inadequate health education, and disparities in socioeconomic status contribute to differences in health outcomes, including those for NCDs [12, 25]. Identifying these health disparities is critical for addressing more vulnerable groups’ specific challenges and unpacking the underlying social determinants of health. Available data suggest that populations living in peri-urban and rural areas have limited awareness about their blood pressure status [16]. Moreover, rural communities tend to be underdiagnosed due to limited disease awareness and limited access to diagnostic or laboratory testing facilities [25]. Studying NCD disease prevalence in rural areas and understanding their behavioral and sociodemographic risk factors is crucial to quantifying, understanding, and beginning to address the needs of these peripheral/underserved populations.

This study attempts to fill these gaps in knowledge and examine the differences with NCD and its risk factors. The findings of our research will be helpful for policymakers and researchers to quantify the NCD burden in the peri-urban and rural communities, where access to NCD services tends to be limited or inadequate [26]. Additionally, these insights may help inform the allocation of resources and the design effective programs and policies to reduce the burden of NCDs in Uganda and other similar LMICs.

## Materials and methods

### Study design, settings, and participants

This population-based cross-sectional survey was conducted at the Iganga-Mayuge Health and Demographic Surveillance Site (IM-HDSS) in Eastern Uganda. Data collection took place between November 2017 and June 2018. The study site includes sixty-five villages from seven sub-countries. IM-HDSS staff members visit the study site twice a year to collect demographic information and vital event data. The study site was established in 2004 [26]. Every village, household, and resident of the area receives a unique identification number. The primary demographic information of the study population is stored in a secured database. We used the participants of the database as the source population. From the database of the source population, we randomly selected participants among those at least 18 years of age who lived in the area for at least six months.

The institutional review boards (IRB) of the Makerere University School of Public Health Higher Degrees, Research and Ethics Committee, Uganda National Council of Science and Technology, and Johns Hopkins Bloomberg School of Public Health approved the study protocol.

### Data collection

Data collection took place at each participant’s residence. First, the data collectors obtained informed oral consent, and IRB approved the informed consent process. Then, a shortened version of the STEPS questionnaire was administered using an Android tablet programmed with an open data kit. Participants provided the following information on the questionnaire: demographics, tobacco use, alcohol consumption, dietary intake, and physical activity [27]. After that, trained nurses recorded BP, weight, and height. Data from pregnant women was not collected. BP was recorded three times using the Omicron 5 series upper arm monitors.

For individuals who underwent physical measurements, if their BP readings indicated high BP but not an immediate risk of a severe event (specifically, systolic BP between 140 mmHg and 179 mmHg and diastolic BP below 120 mmHg), the research team educated them about the significance of maintaining optimal BP levels. In contrast, when a participant exhibited signs of a hypertensive crisis (i.e., having a systolic BP of 180 mmHg or higher or a diastolic BP of 120 mmHg or higher), appropriate arrangements were put in place to transport them to the nearest healthcare facility, provided they consented. It is worth noting that, as per the guidelines established by Uganda’s Ministry of Health, government healthcare facilities are obligated to offer free healthcare services, including medication for hypertension [28].

For anthropometric (i.e., body height and weight) measurements, participants were instructed to stand upright without wearing shoes and with light-weight clothes. Height and body weight were measured twice to the nearest 0.1 centimeters and kilogram, respectively.

### Outcomes

In assessing BP, the final average systolic and diastolic BP estimates were determined using the mean of three readings. Participants were categorized as either hypertensive or pre-hypertensive, following the guidelines set forth by the WHO-International Society of Hypertension (WHO-ISH) [29, 30].

In accordance with the STEPS analysis guide [27], indicator variables were generated to assess NCD risk factors. We used the following indicators: tobacco smoking (current & daily); smokeless tobacco using (current & daily); tobacco using (current & daily); current drinking; heavy episodic drinking; low fruit & vegetable consumption; insufficient physical activity; always/often add salt while eating; always/often add salt while cooking; body weight; prehypertension; and hypertension.

To account for the limited prevalence of tobacco smokers and users of smokeless tobacco, we utilized "current tobacco use (any type)" in the regression models. "Current drinkers" were individuals who consumed alcohol within the past 30 days. "Heavy episodic drinking" was defined as the consumption of six or more standard alcoholic drinks on a single occasion within the past 30 days. "Low fruit and vegetable consumption" refers to the consumption of fewer than five servings of fruits and/or vegetables on average per day in a week.

The analysis followed the Global Physical Activity Questionnaire (GPAQ) guide [19] to establish indicators for physical activity levels. Minutes spent engaging in physical activities were multiplied by a metabolic equivalent (MET) value based on the type of activity, using 8 MET for vigorous-intensity activities and 4 MET for moderate-intensity activities. "Insufficient physical activity" was characterized by individuals who failed to meet any of the following criteria: 150 minutes of moderate-intensity physical activity per week, 75 minutes of vigorous-intensity physical activity per week, or an equivalent combination of moderate- and vigorous-intensity physical activity that amounted to at least 600 metabolic equivalent-minutes per week [31].

Final estimates for height and weight were obtained by averaging two separate measurements. Subsequently, we calculated Body Mass Index (BMI), expressed in kilograms per square meter (kg/m^2^). BMI was then grouped into the following categories [32]: underweight (below18.5 kg/m^2^), normal weight (18.5 and less than 25 kg/m^2^), overweight (25 and less than 30 kg/m^2^), and obese (greater than 30 kg/m^2^).

Hypertension was defined as having a systolic BP equal to or greater than 140 mmHg, a diastolic BP equal to or greater than 90 mmHg, or being on anti-hypertensive medication during the data collection period. Prehypertension was defined as having a systolic BP ranging from 120 to 139 mmHg and/or a diastolic BP ranging from 80 to 89 mmHg [29, 30].

### Exposures

For equity distribution, we compared differences in the prevalence of NCD risk factor indicators for the following exposure variables: sex (i.e., male vs. female), location (i.e., peri-urban vs. rural), wealth status (i.e., ’bottom 40% vs. upper 60%’), and education (i.e., no education vs. primary or more). Wealth index score was generated from principal component analysis of household assets, and it was then stratified into quintiles (i.e., lowest, lower, middle, richer, and richest).

### Data analysis

First, we reported the sociodemographic characteristics (i.e., age, sex, or education level) of respondents. We described continuous variables (e.g., age) with mean and standard error (SE). For categorical variables (e.g., education level), we reported weighted percentages (%) and unweighted numbers (n). We reported each indicator’s prevalence rate with 95% confidence intervals (CIs). To compare different sociodemographic groups, we used Poisson regression. We reported unadjusted and adjusted prevalence ratios (PR) with 95% CIs. We adjusted for age, sex, location, and education in the adjusted analysis. The analyses were conducted using Stata 15.0 (College Station, TX, USA).

### Sample size

We used stratified random sampling to calculate sample size. Participants were drawn from males and females of 18-29-, 30-44-, 45-59- and 60+-year-old age groups (i.e., eight age-sex strata). To have the 50% prevalence rate, a 5% margin-of-error (δ = 0. 05), 80% power, and a 5% type-I error (α = 0. 05) for each stratum, we needed 385 participants. The 50% prevalence rate was used to have the highest possible number of sample size with adequate power. We inflated the sample size in two ways. First, we adjusted for the refusal and outmigration and inflated by 15%. Then, we inflated it by an additional 20% to account for the overall lost-to-follow-up; therefore, we needed a sample of 567 for each stratum and 4536 total required sample.

## Results

Table 1 shows the sociodemographic characteristics of respondents according to sex (n = 3220). The mean age of the participants was 35.3 (SE: 0.1) years (35.7 (SE: 0.15) years among females and 34.9 (SE: 0.15) years among males). The proportion of people without formal education was 9.1% (13.2% among females and 4.6% among males). About two-thirds of the respondents were currently married (61.8%) or were residing in rural regions (59.3%). The distribution of wealth status (males vs females, poorest: 19.2% vs 21.5%, and richest: 16.8% vs 13.5%) was also similar. A majority of the respondents were Muslims (55.3%).

**Table 1:** Demographic characteristics of study participants; Iganga-Mayuge, Uganda, % (n)^1^.

Variable		Overall	Female	Male
		(n = 3220)	(n = 1716)	(n = 1404)
**Age (in year)**	Mean (SE)	35.3 (0.10)	35.7 (0.15)	34.9 (0.15)
	18–29	46.9 (739)	46.2 (374)	47.6 (365)
	30–44	29.3 (767)	29.2 (392)	29.5 (375)
	45–59	15.6 (826)	15.7 (465)	15.5 (361)
	60+	8.1 (888)	8.8 (485)	7.4 (403)
**Education level**	No education	9.1 (590)	13.2 (448)	4.6 (142)
	Primary	43.9 (1526)	44.1 (791)	43.8 (735)
	Secondary	38.2 (877)	34.7 (384)	42 (493)
	More than secondary	8.8 (227)	7.9 (93)	9.6 (134)
**Marital status**	Never married	27.6 (441)	19.8 (161)	36 (280)
	Married	61.8 (2100)	63.9 (991)	59.6 (1109)
	Divorced/separated	5.5 (243)	7.1 (162)	3.7 (81)
	Widowed	5.1 (436)	9.2 (402)	.8 (34)
**Wealth index**	Lowest	20.4 (656)	19.2 (341)	21.5 (315)
	Lower	20.7 (534)	19.2 (269)	22.2 (265)
	Middle	21.5 (590)	22.1 (318)	20.9 (272)
	Higher	22.3 (519)	22.7 (287)	21.8 (232)
	Highest	15.2 (332)	16.8 (193)	13.5 (139)
**Location**	Rural	59.3 (2102)	59.9 (1125)	58.6 (977)
	Per-urban	40.7 (1117)	40.1 (590)	41.4 (527)
**Religion**	Muslim	55.3 (1589)	54.7 (822)	56 (767)
	Protestant	30 (945)	30.3 (500)	29.7 (445)
	Catholic	8.1 (256)	8.2 (137)	7.9 (119)
	Pentacostal	5.4 (146)	5.3 (83)	5.5 (63)
	Other Christian	1.2 (31)	1.6 (19)	(12)

1. Weighted column percentage and unweighted numbers unless otherwise specified.

In Table 2, we reported the overall and sex-stratified prevalence of NCD indicators along with the association of sex with those indicators. Males had a higher prevalence of most behavioral risk factors than females, including for the tobacco and alcohol indicators. Overall prevalence of current tobacco use was 4.2% (95% CI: 3.4%-5.3%); 0.7% (95% CI: 0.4%-1.1%) among females, and 8.4% (95% CI: 6.3%-9.1%) among males; the adjusted PR (APR) was 13.4 (95% CI: 7.9–22.7) times as high among males than among females. The respective prevalence of poor physical activity, overweight, and obesity were lower among males than females. The overall, female, and male prevalence of hypertension were 17.1% (95% CI: 15.8%-18.5%), 17.6% (95% CI: 15.8%-19.6%), and 16.6% (95% CI: 14.7%-18.7%), respectively.

**Table 2:** Weighted estimates of non-communicable disease risk factors by sex.

Variable	Overall, % (95% CI)	Female, % (95% CI)	Male, % (95% CI)	CPR	APR
**Current tobacco smoker**	4.0 (3.4,4.8)	0.7 (0.4,1.1)	7.6 (6.3,9.1)	11.6*** [6.7,20.0]	12.8*** [7.4,22.3]
**Daily tobacco smoker**	2.6 (2.1,3.2)	0.3 (0.2,0.6)	5.0 (3.9,6.3)	15.1*** [7.4,30.7]	17.0*** [8.4,34.6]
**Current smokeless tobacco user**	0.7 (0.5,1.2)	0.1 (0.1,0.3)	1.4 (0.8,2.4)	10.5*** [3.9,28.5]	10.5*** [4.1,27.0]
**Daily smokeless tobacco user**	0.3 (0.2,0.7)	0.1 (0.0,0.2)	0.6 (0.3,1.4)	9.2** [2.1,39.3]	9.5*** [2.5,35.5]
**Current tobacco user**	4.4 (3.7,5.3)	0.7 (0.4,1.1)	8.4 (7.0,10.0)	12.2*** [7.2,20.6]	13.4*** [7.9,22.7]
**Current drinker**	7.7 (6.7,8.8)	4.6 (3.6,5.8)	11.1 (9.4,13.0)	2.4*** [1.8,3.2]	2.4*** [1.8,3.2]
**Heavy episodic drinker**	6.6 (5.7,7.7)	4.0 (3.0,5.2)	9.4 (7.8,11.2)	2.3*** [1.7,3.2]	2.3*** [1.6,3.2]
**Low fruit & vegetable consumption**	98.5 (97.9,98.9)	98.1 (97.1,98.7)	98.9 (98.1,99.3)	1.0 [1.0,1.0]	1.0 [1.0,1.0]
**Always/often add salt while eating**	4.6 (3.7,5.6)	4.1 (3.1,5.6)	5.0 (3.9,6.6)	1.2 [0.8,1.8]	1.2 [0.8,1.9]
**Always/often add salt while cooking**	46.8 (44.7,48.9)	45.5 (42.6,48.5)	48.1 (45.0,51.1)	1.1 [1.0,1.2]	1.0 [0.9,1.1]
**Always eat processed foods high in salt**	10.2 (8.9,11.6)	7.1 (5.7,8.9)	13.4 (11.4,15.8)	1.9*** [1.4,2.5]	1.8*** [1.4,2.4]
**Poor physical activity**	8.0 (7.0,9.1)	9.9 (8.4,11.7)	5.9 (4.7,7.3)	0.6*** [0.4,0.8]	0.6*** [0.5,0.8]
**Underweight**	2.1 (1.6,2.7)	2.0 (1.4,2.8)	2.3 (1.6,3.3)	1.2 [0.7,1.9]	1.3 [0.8,2.2]
**Overweight**	26.9 (25.0,28.8)	30.7 (28.0,33.6)	23.1 (20.7,25.7)	0.8*** [0.7,0.9]	0.7*** [0.6,0.9]
**Obese**	9.3 (8.2,10.5)	14.7 (12.8,16.8)	4.1 (3.1,5.2)	0.3*** [0.2,0.4]	0.3*** [0.2,0.3]
**Prehypertension**	58.9 (56.5,61.3)	50.8 (47.3,54.3)	66.8 (63.4,69.9)	1.3*** [1.2,1.4]	1.3*** [1.2,1.4]
**Hypertension**	17.1 (15.8,18.5)	17.6 (15.8,19.6)	16.6 (14.7,18.7)	0.9 [0.8,1.1]	1.0 [0.9,1.2]

Abbreviations: CPR: Crude prevalence ratio, APR: Adjusted prevalence ratio; CI: Confidence interval

*: p<0.05

**: p<0.01; &

***: p<0.001

Regarding differences in the burden of non-communicable diseases or their risk factors by location (Table 3), the prevalence of alcohol indicators was higher in peri-urban than in rural regions. The prevalence of current drinkers was 10.4% (95% CI: 8.6%-12.6%) in peri-urban areas and 5.8% (95% CI: 4.8%-7.1%) in rural regions; the APR was 1.8 (95% CI: 1.3–2.4). Prevalence of heavy episodic drinking was 9.9% (95% CI: 8.2%-12.1%) in peri-urban regions and 4.3% (95% CI: 3.4%-5.5%) in rural areas, the APR was 2.2 (95% CI: 1.6–3.1). Peri-urban regions had a significantly higher prevalence of the following other indicators than rural regions: current tobacco use (APR: 1.5, 95% CI: 1.1–2.1), always/often adding salt while cooking (APR: 1.2, 95% CI: 1.1–1.4), always eat processed foods high in salt (APR: 2.1, 95% CI: 1.6–2.8), poor physical activity (APR: 1.5, 95% CI: 1.1–2.0), obesity (APR: 1.5, 95% CI: 1.1–1.9), and hypertension (APR: 1.2, 95% CI: 1.0–1.5).

**Table 3:** Weighted estimates of non-communicable disease risk factors by location.

Variable	Rural, % (95% CI)	Peri-urban, % (95% CI)	CPR	APR
**Current tobacco smoker**	4.0 (3.2,5.1)	4.0 (3.0,5.3)	1.0 [0.7,1.4]	1.4 [1.0,2.0]
**Daily tobacco smoker**	2.8 (2.1,3.7)	2.3 (1.6,3.3)	0.8 [0.5,1.3]	1.3 [0.8,2.1]
**Current smokeless tobacco user**	0.5 (0.2,0.9)	1.2 (0.6,2.3)	2.6 [1.0,6.9]	2.8 [0.9,9.0]
**Daily smokeless tobacco user**	0.2 (0.1,0.4)	0.6 (0.2,1.6)	3.7 [0.9,15.4]	4.1 [0.9,19.0]
**Current tobacco user**	4.2 (3.4,5.3)	4.7 (3.6,6.1)	1.1 [0.8,1.6]	1.5* [1.1,2.1]
**Current drinker**	5.8 (4.8,7.1)	10.4 (8.6,12.6)	1.8*** [1.4,2.3]	1.8*** [1.3,2.4]
**Heavy episodic drinker**	4.3 (3.4,5.5)	9.9 (8.2,12.1)	2.3*** [1.7,3.1]	2.2*** [1.6,3.1]
**Low fruit & vegetable consumption**	98.5 (97.8,99.1)	98.3 (97.3,99.0)	1.0 [1.0,1.0]	1.0 [1.0,1.0]
**Always/often add salt while eating**	5.1 (4.0,6.5)	3.8 (2.6,5.3)	0.7 [0.5,1.1]	0.7 [0.4,1.0]
**Always/often add salt while cooking**	39.9 (37.3,42.6)	56.8 (53.5,60.1)	1.4*** [1.3,1.6]	1.2*** [1.1,1.4]
**Always eat processed foods high in salt**	6.6 (5.3,8.2)	15.4 (13.0,18.1)	2.3*** [1.8,3.1]	2.1*** [1.6,2.8]
**Poor physical activity**	6.9 (5.8,8.2)	9.6 (7.8,11.6)	1.4* [1.1,1.8]	1.5** [1.1,2.0]
**Underweight**	1.9 (1.3,2.6)	2.5 (1.7,3.6)	1.3 [0.8,2.2]	1.7 [1.0,3.2]
**Overweight**	27.8 (25.4,30.3)	25.5 (22.7,28.6)	0.9 [0.8,1.1]	0.9 [0.8,1.0]
**Obese**	7.6 (6.4,9.0)	11.7 (9.9,13.9)	1.5*** [1.2,2.0]	1.5** [1.1,1.9]
**Prehypertension**	55.1 (51.9,58.2)	64.4 (60.5,68.1)	1.2*** [1.1,1.3]	1.2*** [1.1,1.3]
**Hypertension**	17.5 (15.9,19.3)	16.4 (14.3,18.8)	0.9 [0.8,1.1]	1.2* [1.0,1.5]

Abbreviations: CPR: Crude prevalence ratio, APR: Adjusted prevalence ratio; CI: Confidence interval

*: p<0.05

**: p<0.01; &

***: p<0.001

When we compared participants according to wealth, during the comparison of ’bottom 40%’ to ’top 60%’ (Table 4), most indicators did not differ. For instance, the prevalence of poor physical activity was 6.5% (95% CI: 5.2–8.2) among the ’bottom 40%’ and 9.3% (95% CI: 7.7–11.3) among the ’top 60%’. Similarly, the prevalence of obesity was 5.7% (95% CI: 4.3%-7.4%) among the ’bottom 40%’ and 12.4% (95% CI: 10.7%-14.4%) among the ’top 60%’. Compared to the ’bottom 40%’, only three indicators were significantly higher among ’top 60%’ people: always or often add salt while cooking (APR: 1.3, 95% CI: 1.1–1.4), poor physical activity (APR: 1.4, 95% CI: 1.0–1.9), and obesity (APR: 1.8, 95% CI: 1.3–2.4). Similar findings were observed when we compared ’bottom 20%’ to ’top 20%’ (Table 5).

**Table 4:** Weighted estimates of non-communicable disease risk factors by socioeconomic status (bottom 40 vs upper 60%).

Variable	Bottom 40, % (95% CI)	Top 60, % (95% CI)	CPR	APR
**Current tobacco smoker**	4.7 (3.6,6.2)	3.2 (2.4,4.3)	0.7 [0.5,1.0]	0.8 [0.6,1.2]
**Daily tobacco smoker**	3.4 (2.5,4.8)	1.6 (1.1,2.5)	0.5** [0.3,0.8]	0.7 [0.4,1.1]
**Current smokeless tobacco user**	0.4 (0.1,1.1)	0.7 (0.3,1.5)	1.7 [0.4,6.6]	1.5 [0.2,9.8]
**Daily smokeless tobacco user**	0.3 (0.1,1.1)	0.2 (0.0,0.9)	0.5 [0.1,4.7]	0.2 [0.0,2.6]
**Current tobacco user**	4.9 (3.7,6.5)	3.5 (2.7,4.7)	0.7 [0.5,1.1]	0.9 [0.6,1.3]
**Current drinker**	6.7 (5.3,8.5)	7.9 (6.4,9.6)	1.2 [0.9,1.6]	1.0 [0.7,1.4]
**Heavy episodic drinker**	5.1 (3.8,6.7)	7.0 (5.6,8.7)	1.4 [1.0,2.0]	1.0 [0.7,1.5]
**Low fruit & vegetable consumption**	98.6 (97.4,99.3)	98.6 (97.8,99.1)	1.0 [1.0,1.0]	1.0 [1.0,1.0]
**Always/often add salt while eating**	5.1 (3.6,7.0)	4.8 (3.6,6.3)	0.9 [0.6,1.5]	1.0 [0.6,1.6]
**Always/often add salt while cooking**	36.4 (32.9,40.0)	53.7 (50.7,56.8)	1.5*** [1.3,1.7]	1.3*** [1.1,1.4]
**Always eat processed foods high in salt**	6.7 (5.0,8.9)	12.1 (10.2,14.4)	1.8*** [1.3,2.5]	1.4 [1.0,2.0]
**Poor physical activity**	6.5 (5.2,8.2)	9.3 (7.7,11.3)	1.4* [1.1,1.9]	1.4* [1.0,1.9]
**Underweight**	2.2 (1.6,3.2)	1.8 (1.2,2.8)	0.8 [0.5,1.4]	0.8 [0.5,1.4]
**Overweight**	26.5 (23.4,29.9)	27.1 (24.4,30.0)	1.0 [0.9,1.2]	1.0 [0.9,1.2]
**Obese**	5.7 (4.3,7.4)	12.4 (10.7,14.4)	2.2*** [1.6,3.0]	1.8*** [1.3,2.4]
**Prehypertension**	57.9 (53.7,62.0)	58.6 (55.1,62.1)	1.0 [0.9,1.1]	1.0 [0.9,1.1]
**Hypertension**	18.2 (16.0,20.7)	16.9 (15.0,19.1)	0.9 [0.8,1.1]	1.0 [0.8,1.2]

Abbreviations: CPR: Crude prevalence ratio, APR: Adjusted prevalence ratio; CI: Confidence interval

*: p<0.05

**: p<0.01; &

***: p<0.001

**Table 5:** Weighted estimates of non-communicable disease risk factors by socioeconomic status (bottom 20 vs upper 20%).

Variable	Bottom 20, % (95% CI)	Top 20, % (95% CI)	CPR	APR
**Current tobacco smoker**	5.7 (4.0,8.2)	3.7 (2.1,6.5)	0.6 [0.3,1.3]	1.0 [0.5,2.0]
**Daily tobacco smoker**	4.5 (3.0,6.9)	1.7 (0.7,4.2)	0.4 [0.1,1.0]	0.6 [0.3,1.4]
**Current smokeless tobacco user**	0.3 (0.1,1.0)	0.5 (0.1,3.6)	1.6 [0.2,14.5]	0.3 [0.0,3.5]
**Daily smokeless tobacco user**	0.2 (0.0,0.6)	0.5 (0.1,3.6)	3.4 [0.3,39.3]	1.8 [0.6,5.5]
**Current tobacco user**	5.7 (4.0,8.2)	3.7 (2.1,6.5)	0.6 [0.3,1.3]	1.0 [0.5,2.0]
**Current drinker**	6.7 (4.9,9.2)	10.4 (7.4,14.5)	1.5 [1.0,2.4]	1.0 [0.5,2.0]
**Heavy episodic drinker**	4.7 (3.2,6.7)	9.3 (6.4,13.3)	2.0** [1.2,3.3]	1.1 [0.4,2.7]
**Low fruit & vegetable consumption**	98.9 (97.1,99.5)	98.9 (96.5,99.7)	1.0 [1.0,1.0]	1.0 [1.0,1.0]
**Always/often add salt while eating**	5.6 (3.5,8.6)	6.1 (3.6,10.0)	1.1 [0.6,2.2]	0.6 [0.3,1.2]
**Always/often add salt while cooking**	29.8 (25.3,34.8)	68.6 (62.7,73.9)	2.3*** [1.9,2.7]	2.1*** [1.7,2.8]
**Always eat processed foods high in salt**	6.2 (4.0,9.6)	19.8 (15.3,25.4)	3.2*** [1.9,5.3]	2.5** [1.3,5.1]
**Poor physical activity**	7.7 (5.7,10.3)	14.2 (10.3,19.2)	1.8** [1.2,2.8]	2.1* [1.0,4.5]
**Underweight**	2.6 (1.7,4.0)	2.2 (1.0,4.8)	0.8 [0.3,2.1]	2.4 [0.3,21.3]
**Overweight**	23.0 (19.1,27.4)	30.1 (24.7,36.0)	1.3* [1.0,1.7]	1.2 [0.8,1.9]
**Obese**	5.3 (3.6,7.7)	17.4 (13.4,22.2)	3.3*** [2.1,5.2]	2.4** [1.3,4.6]
**Prehypertension**	53.2 (47.3,59.1)	56.5 (49.1,63.6)	0.9 [0.7,1.2]	1.1 [0.8,1.4]
**Hypertension**	20.9 (17.7,24.5)	18.8 (14.9,23.5)	0.9 [0.7,1.2]	1.4 [0.9,2.0]

Abbreviations: CPR: Crude prevalence ratio, APR: Adjusted prevalence ratio; CI: Confidence interval

*: p<0.05

**: p<0.01; &

***: p<0.001

The findings were similar when we compared people by education level (Table 6); compared to people without any formal education, people with primary/above education had a significantly higher prevalence of always/often adding salt while cooking (APR: 1.4, 95% CI: 1.1–1.7) and obesity (APR: 1.6, 95% CI: 1.1–2.4). On the other hand, people with primary/above education had a significantly lower prevalence of poor physical activity (APR: 0.6, 95% CI: 0.5–0.9) and underweight (APR: 0.6, 95% CI: 0.4–0.9).

**Table 6:** Weighted estimates of non-communicable disease risk factors by education.

Variable	None, % (95% CI)	Primary/more, % (95% CI)	CPR	APR
**Current tobacco smoker**	4.7 (3.1,7.0)	4.0 (3.3,4.8)	0.8 [0.5,1.3]	0.8 [0.5,1.2]
**Daily tobacco smoker**	2.9 (1.7,4.7)	2.5 (2.0,3.2)	0.9 [0.5,1.6]	0.8 [0.5,1.5]
**Current smokeless tobacco user**	0.4 (0.1,1.2)	0.8 (0.5,1.3)	2.1 [0.6,7.5]	1.1 [0.3,3.7]
**Daily smokeless tobacco user**	0.3 (0.1,1.2)	0.3 (0.1,0.8)	1.2 [0.2,6.3]	0.6 [0.1,3.3]
**Current tobacco user**	4.9 (3.3,7.2)	4.4 (3.6,5.3)	0.9 [0.6,1.4]	0.8 [0.5,1.2]
**Current drinker**	7.3 (5.1,10.5)	7.7 (6.7,8.9)	1.1 [0.7,1.6]	1.0 [0.7,1.6]
**Heavy episodic drinker**	4.8 (3.0,7.8)	6.8 (5.8,7.9)	1.4 [0.8,2.3]	1.3 [0.8,2.3]
**Low fruit & vegetable consumption**	98.3 (96.5,99.2)	98.5 (97.8,98.9)	1.0 [1.0,1.0]	1.0 [1.0,1.0]
**Always/often add salt while eating**	4.2 (2.4,7.1)	4.6 (3.7,5.7)	1.1 [0.6,2.0]	0.9 [0.4,1.7]
**Always/often add salt while cooking**	25.8 (21.2,31.0)	48.8 (46.6,51.1)	1.9*** [1.6,2.3]	1.4** [1.1,1.7]
**Always eat processed foods high in salt**	3.9 (2.0,7.4)	10.8 (9.4,12.3)	2.8** [1.4,5.5]	1.5 [0.7,2.9]
**Poor physical activity**	17.8 (14.6,21.6)	7.0 (5.9,8.2)	0.4*** [0.3,0.5]	0.6** [0.5,0.9]
**Underweight**	5.0 (3.7,6.9)	1.8 (1.3,2.5)	0.4*** [0.2,0.6]	0.6* [0.4,0.9]
**Overweight**	28.3 (23.7,33.3)	26.7 (24.8,28.8)	0.9 [0.8,1.1]	1.1 [0.9,1.4]
**Obese**	10.5 (7.5,14.4)	9.2 (8.1,10.5)	0.9 [0.6,1.2]	1.6* [1.1,2.4]
**Prehypertension**	42.0 (37.0, 47.2)	49.6 (47.3, 51.8)	1.2* [1.0,1.3]	0.9 [0.8,1.1]
**Hypertension**	32.9 (28.7,37.4)	15.5 (14.1,17.0)	0.5*** [0.4,0.6]	1.0 [0.9,1.2]

Abbreviations: CPR: Crude prevalence ratio, APR: Adjusted prevalence ratio; CI: Confidence interval

*: p<0.05

**: p<0.01; &

***: p<0.001

## Discussion

In this study, we investigated the sociodemographic and socioeconomic differences related to NCD indicators in a rural and peri-urban community in Uganda. Although we found gender differences in the prevalence of most NCD indicators, when we compared them by location, wealth, or education, we did not find significant differences for most of them. Our study adds to the growing body of literature testing differences in the burden of NCD risk factors in Uganda and other similar LMICs.

The sex or socioeconomic differences we observed here were reported by multiple earlier studies in other LMICs [33–36], including studies from sub-Saharan Africa [24, 33, 37]. For instance, similar to our findings, a study by Sreeramareddy and Acharya analyzed data from 22 sub-Saharan African countries and found a higher prevalence of tobacco smoking among people with male gender, low education, low wealth, and older age [33]. However, when we reported the prevalence, the overall prevalence of current tobacco smoking or use we observed here was lower than the STEPS 2014 report [16]. This may result from the fact that STEPS 2014 was a nationally representative survey, while our survey was conducted in a community within one region. Nevertheless, similar to the pattern observed by the STEPS 2014 or GATS 2013 report, we found a higher prevalence of tobacco use or smoking among males than females. Societal or familial norms prohibit women from smoking in many developing countries [38]. Considering the impact of negative impacts of tobacco consumption, males need more awareness about it; a sex-specific approach to increasing awareness among men may also reduce the prevalence among them. We did not observe the difference in prevalence of tobacco use for most other variables. From a public health standpoint, any form of tobacco is harmful to humans; tobacco consumption is alone responsible for 6 million global deaths, and smoking cessation would have a significant impact on the health system [39]. Uganda’s Tobacco Control Act of 2015 aims to regulate tobacco use. It includes the implementation of smoke-free places; tobacco advertisement, promotion, and sponsorship; package and labeling of packages; and sales restrictions [40]. Although it would take a long time to see the impact of this type of legislation on tobacco control behaviors, data from Uganda and multiple other sub-Saharan African countries indicate that the prevalence of tobacco use is declining in this region [33]; this is one of the welcome aspects of tobacco consumption in this region.

We also observed the association of sex and location with alcohol consumption. This is also similar to other studies from Uganda and other sub-Saharan African countries [21, 37, 41]. Men and peri-urban residents had a higher prevalence of alcohol consumption than women and rural residents. These may result from differences in access to alcohol. Excessive alcohol consumption is also a significant risk factor for many NCDs [2–5]. The proportion we observed is substantially lower than STEPS 2014 [16]. This may occur due to a considerably higher proportion of Muslims among our study population compared to the overall country; due to religious restrictions, Muslims consume less alcohol, which may have contributed to this difference [42]. Moreover, our sample contains a predominantly rural population, which may also contribute to observed differences. In our study, most respondents did not consume an adequate amount of fruit and vegetables. The prevalence rate of this indicator was high regardless of sex, location, wealth, or location. It increases the risks for other NCDs, such as hypertension, dyslipidemia, and overweight/obesity [17, 43]. Cultural dietary patterns, limited knowledge about optimal nutrient balance, limited availability, and increased urbanization could all contribute [17, 44]. In addition to raising awareness about the importance of fruit and vegetable consumption, it is essential to design and formulate policies to increase their availability [12].

We observed that more than one-third of respondents had a higher-than-normal body weight (i.e., overweight or obese). It differed by most characteristics, including sex, wealth, and education. Prior studies have reported that females are less likely to engage in physical exercise, which may contribute to sex differences in overweight or obesity; our findings were congruent with lower levels of physical activity among women Peri-urban residents and people with higher socioeconomic status following a more sedentary lifestyle that can also explain the difference by location, wealth, or education [11, 20, 21]. It is essential to raise awareness about increasing physical activity and decreasing the burden of overweight/obesity in these population groups.

Although we did not observe differences in the prevalence of hypertension according to most of our studied characteristics, more than half of the people had either prehypertension or hypertension. Although the burden of hypertension is lower than the HICs in the US among our study population, the burden of prehypertension was substantially higher [45, 46]. People with prehypertension are also at a higher risk of cardiovascular disease, including hypertension [30]. A significant proportion of people might remain unaware of their BP levels because they may have never measured it in their lives. This high burden indicates that a large proportion of people in this country need to take adequate measures to reduce the burden of hypertension. Moreover, the risk factors and prevention strategies for hypertension and most other NCDs (e.g., diabetes, obesity, and dyslipidemia) are common, including measures related to physical activity, salt, and alcohol consumption [12, 30, 47]. Therefore, the prevention of these conditions would have a simultaneous impact on the burden of the health system.

This study has several notable strengths. We investigated these risk factors using a large population-based sample. Trained interviewers collected data using a standardized and validated questionnaire. The response rate was high, and non-response was minimized by visiting the participant’s household at least three times at varying times to ensure the presence of participants.

Our study has some limitations. First, as this was a cross-sectional study, the data on factors and outcomes studied here were collected simultaneously; therefore, causality among some sociodemographic factors and NCD indicators cannot be established. As the data are self-reported, we cannot rule out recall bias or measurement error about variables like salt, vegetable, or alcohol consumption. Moreover, people may also report some responses to please interviewers (i.e., social desirability bias), especially about indicators related to alcohol consumption. Lastly, standard guidelines recommend recording multiple longitudinal measurements of BP in a clinical setting to diagnose hypertension; however, we recorded BP on a single day, which may have also caused some nondifferential misclassification [30].

## Conclusions

Our study in Iganga Mayuge revealed noteworthy variations in key NCD indicators based on various demographic and socioeconomic factors, particularly emphasizing gender differences. These findings offer crucial insights for policymakers and public health practitioners, providing an understanding of the NCD burden. The awareness generated can inform the design of effective, targeted programs tailored to address the distinctive health challenges in Iganga Mayuge and comparable peri-urban areas in Uganda. Ongoing research and collaborative efforts are imperative to refine interventions and enhance overall health outcomes in these communities.

## Supporting information

S1 Checklist Inclusivity in global research. (DOCX)
